# A novel method of calculating stroke volume using point-of-care echocardiography

**DOI:** 10.1186/s12947-020-00219-w

**Published:** 2020-08-20

**Authors:** Ehson Aligholizadeh, William Teeter, Rajan Patel, Peter Hu, Syeda Fatima, Shiming Yang, Gautam Ramani, Sami Safadi, Peter Olivieri, Thomas Scalea, Sarah Murthi

**Affiliations:** 1 Division of Trauma and Surgical Critical Care, Department of Surgery, University of Maryland School of Medicine, 22 South Greene St, Baltimore, MD 21201 USA; 2 University of Maryland School of Medicine, Anesthesiology, 22 South Greene St, Baltimore, MD 21201 USA; 3 Division of Cardiovascular Medicine, University of Maryland School of Medicine, 22 South Greene St, Baltimore, MD 21201 USA; 4 University of Maryland School of Medicine, Pulmonary and Critical Care Medicine, 22 South Greene St, Baltimore, MD 21201 USA; 5 University of Maryland Baltimore Washington Medical Center, Pulmonary and Critical Care, 301 Hospital Dr, Glen Burnie, MD 21061 USA

**Keywords:** Echocardiography, POCUS, Hemodynamic monitoring, Cardiac output, Fluid resuscitation

## Abstract

**Background:**

Point-of-care transthoracic echocardiography (POC-TTE) is essential in shock management, allowing for stroke volume (SV) and cardiac output (CO) estimation using left ventricular outflow tract diameter (LVOTD) and left ventricular velocity time integral (VTI). Since LVOTD is difficult to obtain and error-prone, the body surface area (BSA) or a modified BSA (mBSA) is sometimes used as a surrogate (LVOTD^BSA^, LVOTD^mBSA^). Currently, no models of LVOTD based on patient characteristics exist nor have BSA-based alternatives been validated.

**Methods:**

Focused rapid echocardiographic evaluations (FREEs) performed in intensive care unit patients over a 3-year period were reviewed. The age, sex, height, and weight were recorded. Human expert measurement of LVOTD (LVOTD^HEM^) was performed. An epsilon-support vector regression was used to derive a computer model of the predicted LVOTD (LVOTD^CM^). Training, testing, and validation were completed. Pearson coefficient and Bland-Altman were used to assess correlation and agreement.

**Results:**

Two hundred eighty-seven TTEs with ideal images of the LVOT were identified. LVOTD^CM^ was the best method of SV measurement, with a correlation of 0.87. LVOTD^mBSA^ and LVOTD^BSA^ had correlations of 0.71 and 0.49 respectively. Root mean square error for LVOTD^CM^, LVOTD^mBSA^, and LVOTD^BSA^ respectively were 13.3, 37.0, and 26.4. Bland-Altman for LVOTD^CM^ demonstrated a bias of 5.2. LVOTD^CM^ model was used in a separate validation set of 116 ideal images yielding a linear correlation of 0.83 between SV^HEM^ and SV^CM^. Bland Altman analysis for SV^CM^ had a bias of 2.3 with limits of agreement (LOAs) of − 24 and 29, a percent error (PE) of 34% and a root mean square error (RMSE) of 13.9.

**Conclusions:**

A computer model may allow for SV and CO measurement when the LVOTD cannot be assessed. Further study is needed to assess the accuracy of the model in various patient populations and in comparison to the gold standard pulmonary artery catheter. The LVOTD^CM^ is more accurate with less error compared to BSA-based methods, however there is still a percentage error of 33%. BSA should not be used as a surrogate measure of LVOTD. Once validated and improved this model may improve feasibility and allow hemodynamic monitoring via POC-TTE once it is validated.

## Background

Accurate hemodynamic monitoring is vital in the diagnosis and management of critically ill patients, allowing both identification of underlying pathology and selection of appropriate resuscitative therapy [1]. The best method for monitoring remains controversial, explaining the many options available to the clinician. The gold-standard method for such monitoring has long been calculation of cardiac output (CO) by thermodilution using pulmonary artery catheterization (PAC). However, its use has been limited due to many clinicians’ inability to accurately interpret the results [2], its invasive nature, and the lack of a clear survival benefit [3]. These limitations have led to other methods including less-invasive arterial waveform morphomics, radionuclide imaging, and point-of-care transthoracic echocardiography (POC-TTE).

Unlike other methods of hemodynamic monitoring, POC-TTE is non-invasive, allows serial measurements with relatively low resource utilization, and does not expose the patient to ionizing radiation. Further, it is useful for assessing cardiac structure, function, fluid status, as well as hemodynamic monitoring through estimation of stroke volume (SV) and cardiac output (CO) using the SV x the heart rate (HR) [4–6]. Echo derivation of SV, including measurement of the left ventricular outflow tract diameter (LVOTD) and left ventricular outflow tract velocity time integral (VTI), has shown strong correlation and moderate agreement in both surgical and non-surgical patients when compared to PAC [7]. Using TTE, estimation of hemodynamic variables including CO and systemic vascular resistance (SVR) relies on a relatively simple calculation requiring the patient’s mean arterial pressure, heart rate, height, weight, as well as LVOT VTI and LVOTD. The former variables are readily available at the bedside, but the latter require measurement utilizing ultrasonographic Doppler examination.

A significant limitation in the estimation CO and SV by POC-TTE is the inability to measure the LVOTD in about 27.4% of critically ill patients [7, 8]. Fortunately, the LVOTD it is a static anatomic measure in adulthood so it is not needed to trend changes in SV or CO. The LVOT can be estimated using patient’s body surface area (BSA) as a substitute. The BSA and LVOTD differ less than 0.2 units in 75% of patients [9]. To avoid error at values in patient outliers, some groups use modifications of BSA, for example if the BSA is < 1.8m^2^ an LVOTD of 1.8 cm is used and if the BSA is > 2.2m^2^, 2.2 cm is used to estimate the LVOTD [5]. While there is a tradition of using these surrogates, to our knowledge the accuracy in using them to estimate SV and therefore CO has never been determined.

**Fig. 1:**
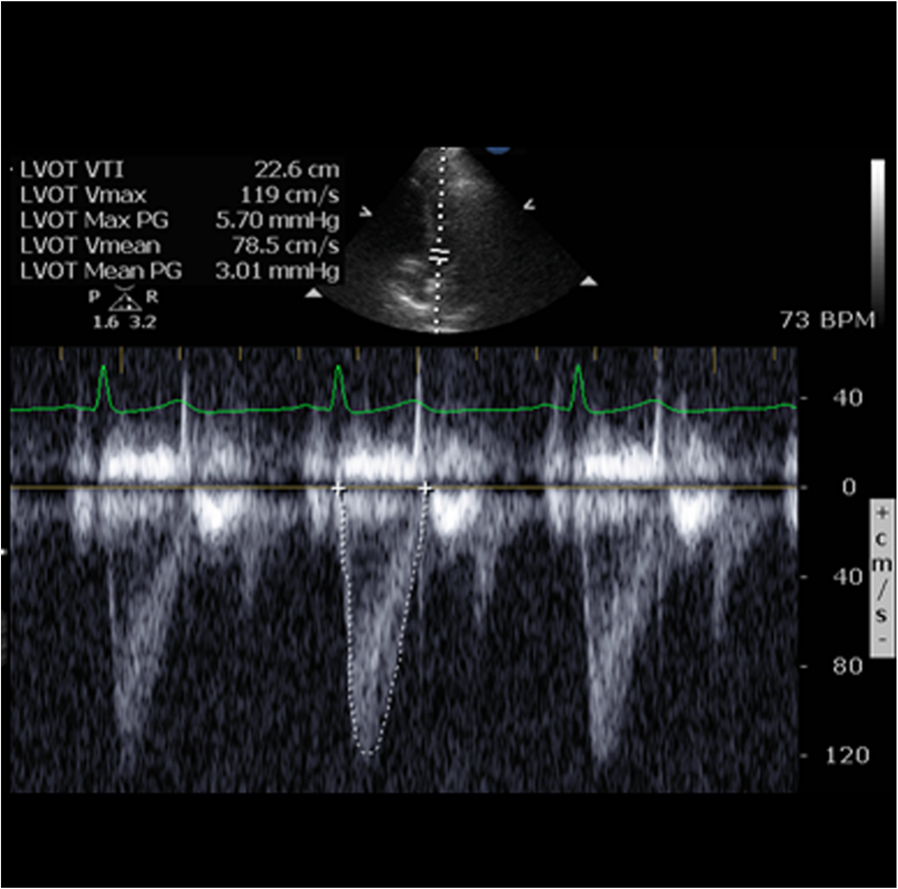
Example image of pulse wave Doppler in the LVOT and measurement of VTI tracing

LVOT VTI is the other echo-derived variable needed and is a dynamic measurement, which changes with fluctuations in SV and directly reflects systolic function. Fortunately, LVOT VTI can be measured in greater than 90% of critically ill patients (Fig. 1) [7]. Therefore, combining the measured and estimated values of LVOTD and LVOT VTI allows calculation of SV at the bedside in most patients, making POC-TTE a viable and reliable alternative to other methods. However, while useful to the clinician, this uncertainty in LVOTD measurement limits its utility, making novel methods for the estimation of cardiac function attractive.

In this study, we aimed to develop an alternative, non-invasive, operator-independent method of SV and CO monitoring using a population-derived, computer-generated model that relies mainly on patient characteristics immediately available, including the patient’s age, sex, height, and weight. We hypothesized that this model would allow for hemodynamic monitoring of critically ill patients with comparable agreement and correlation to TTE and better outcomes than BSA methods. This model will allow for more accurate monitoring in patients whose TTE images are inadequate or difficult to obtain.

## Methods

### Study population

Following approval by our institutional review board, this retrospective, single-center, observational study included data from all ICU patients who had received a focused rapid echocardiographic evaluation (FREE) [4] as part of their clinical care at our academic medical center between January 2015 and April 2018. Any patient less than 18 years of age was excluded. A second population of a patients was abstracted using patients presenting from May 2018 through August 2019. This group of patients was reserved as a separate validation set to further assess the strength of the computer model of LVOTD.

### Image review

**Fig. 2:**
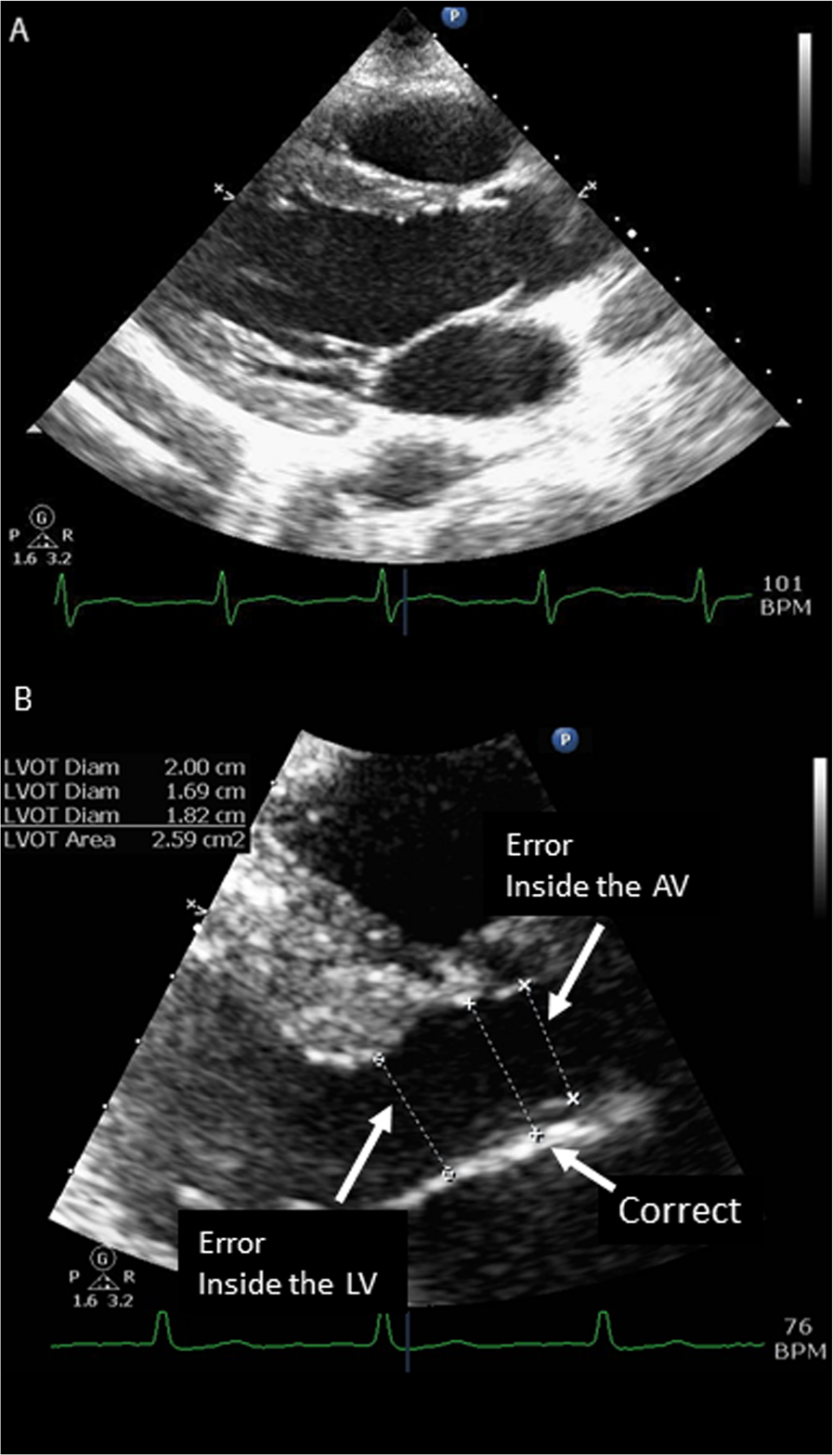
Panel **a**: Example of ideal image obtained in parasternal long at initiation of systole, Panel **b**:ss Magnification of LVOT and examples of common errors in measurement

All studies were reviewed for quality by a registered diagnostic cardiac sonographer (SF) or ultrasound faculty member using the following inclusion criteria: clear visualization of the LVOT and the sinuses of Valsalva, correct measurement of the LVOT just below the aortic annulus, and correct alignment in the parasternal long short axis view (Fig. 2a, b). Patients with non-ideal imaging were excluded. In order to create the most accurate model only images where both aortic valve leaflets could be clearly visualized and in whom the angle of the outflow tract is oriented for precise measurement were included. This is more stringent criteria then we use for clinical interpretation.

Patients were further excluded if they showed evidence of severe aortic stenosis, hypertrophic obstructive cardiomyopathy, or surgically implanted mechanical aortic valve. The sonographer was not blinded to the purpose of the study but was blinded to the patient’s clinical and demographic information.

Twenty ideal images and 10 rejected images were selected randomly using an anonymized study ID by the RANDOM function in Microsoft Excel. These were reviewed by the original reviewer (SF) and an experienced echocardiographer (SM) independently from each other and from the original review. The reviewers were blinded to the results of the original review. There was total agreement amongst the two reviewers on rejection of images and measurement of LVOTD yielded a mean difference of − 0.084 [−.15,-0.02, 95%CI]. The original reviewer produced nearly identical results with total agreement on rejection of images and a mean difference of 0.002 [− 0.062 to 0.066, 95%CI] of measurements of LVOTD.

### Data collection and calculation

FREE was performed on all patients as requested by the clinical care team using a portable cardiovascular ultrasound machine (Philips CX 50, Andover, MA) using a phased array probe. From the POC-TTE images, LVOTD, LVOT VTI, and ejection fraction (EF) were determined. Baseline demographics were obtained retrospectively from the electronic medical record including age, height, weight, gender, race, cardiopulmonary medical history, and surgical history.. Our institution’s policy is to measure the height and weight at hospital admission, these values were used in the calculations.

Stroke volume was calculated using the formula SV = ${\left(\frac{LVOTD}{2}\right)}^2\ast \pi \ast VTI$. Cardiac output was determined by multiplying SV by heart rate (HR). BSA was calculated using BSA = 0.20247 x height (m)^0.725^ x weight (kg)^0.425^. All calculations were performed at the time of clinical evaluation with POC-TTE.

### Data cleaning

For age, height, and weight, data that produced a z score greater than or equal to 3 were excluded from the analysis. All data was then standardized by removing the mean and scaling to unit variance.

### Training, testing, and validation of LVOTD^CM^ model

To derive a computer model estimate of LVOTD (LVOTD^CM^) and subsequently, SV^CM^, the population was split into two sets, 75% for training (*N* = 215), and 25% for testing (*N* = 72). Using these training and testing groups, a gamma for an epsilon-support vector regressor was optimized using a grid search. Shuffle split with 10 splits was used as the cross-validator. The LVOTD^CM^ was then used to calculate the SV^CM^. R, *R*^2^ values, and root mean square error (RMSE) were calculated for *N* = 70. Using identical methods to the original training/testing groups, 507 patients from June 2018 to August 2019 yielded 106 ideal images of LVOTD. The LVOTD^CM^ model was then applied to this validation dataset. Calculations were performed using scikit-learn 0.21.3 (https://scikit-learn.org/). Scikit-learn is suite of machine learning algorithms using Python programming language with a focus on “bringing machine learning to non-specialists using a general-purpose high-level language” described by Pedregosa, Fabian et all in 2011 [10].

### SV estimation methods and statistical comparison

A total of three models for each subject’s SV were used for comparison. In all cases the same VTI obtained from the original POC-TTE was employed. The reference method (SV^HEM^) was the calculated value using the ideal images with human expert measurement (HEM) of the LVOTD^HEM^. LVOTD^CM^ was used to calculate SV^CM^. For SV^BSA^, the BSA in centimeters was used as a surrogate for LVOTD^BSA.^ For SV^mBSA^, if the patient’s calculated BSA was ≤1.8 m^2^, then 1.8 cm was used as the surrogate, if the calculated BSA fell between 1.8–2.2, this value used, and when the calculated BSA was ≥2.2 m^2^, 2.2 cm was used. SV^CM^, SV^BSA^ and SV^mBSA^ were then compared to SV^HEM^ using Bland-Altman analysis.

### Statistical analysis

Pearson coefficient was used to measure correlation and Bland-Altman was used to assess agreement between estimated and measured LVOTD. All statistical analyses unrelated to SV^CM^ modeling were carried out in GraphPad 8.31 (GraphPad Software, La Jolla, CA).

## Results

### Patient characteristics

One thousand one hundred twenty TTEs were reviewed for image adequacy. In order to create the most accurate model, only images where the LVOT could be precisely measured were included. Of the 287 patients with ideal TTE images in the training/testing population, 193 (67%) were from male patients with a mean age of 56 ± 17 years. The demographic breakdown was 139 (48%) Caucasian, 127 (44%) Black, 13 (5%) Hispanic, 2 (1%) Asian, and 6 (2%) unspecified.

### SV measurement and comparison

**Fig. 3:**
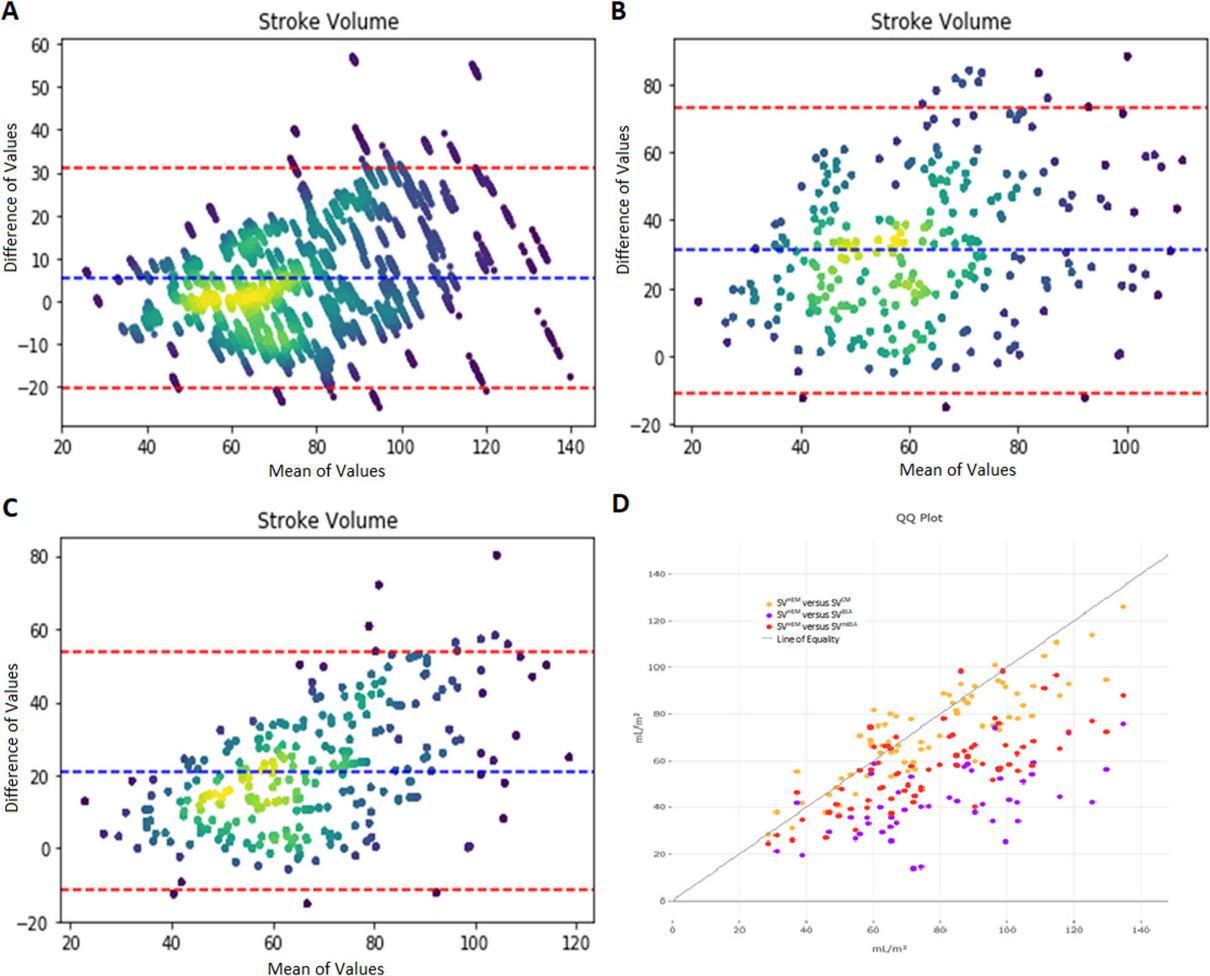
Panel **a**, **b**, and **c**: Bland-Altman plots comparing SV^HEM^ with SV^CM^, SV^BSA^, and SV^mBSA^, respectively. LOA of 95% of the difference between the models will fall between the orange dashed lines. Panel **d**: QQ plot of SV^HEM^ versus SV^CM^, SV^BSA^, SV^mBSA^

Mean SV^HEM^ was 77.5 ± 24 mL and its distribution is shown in Fig. 3. Correlation between SV^HEM^ and SV^CM^ was significant (*r* = 0.87, *p* < 0.0001). Bland-Altman analysis demonstrated a bias of 5.2, limits of agreement (LOAs) of − 19 and + 29 mL/m^2^, and percent error (PE) of 33% (Fig. 3a). Correlation between SV^HEM^ and SV^BSA^ was significant (*r* = 0.49, *p* < 0.0001), and Bland-Altman analysis demonstrated a bias of 29, LOAs of − 15 and 73 mL/m^2^, and PE of 91% (Fig. 3b). Correlation between SV^HEM^ and SV^mBSA^ was significant (*r* = 0.71, *p* < 0.0001), and Bland-Altman analysis demonstrated a bias of 20, LOAs of − 13 and 54 mL/m^2^, and PE of 58% (Fig. 3c). Root mean square error (RMSE) for SV^CM^, SV^BSA^, SV^mBSA^ compared to SV^HEM^ were 13, 37, and 26, respectively.

### Validation

Five hundred seven patients were identified for use in the validation dataset. After review, 116 yielded ideal images adequate for use. Using the derived computer model from the original training and testing dataset, SV^CM^ was validated using this dataset. There were no significant differences in age, gender, race, or height between the Validation group and the Training/Testing groups (all *p* > 0.1). There was significant difference between the Training/Testing group and the Validation group in patient weight (84 ± 24 kg vs. 89 ± 22 kg; *p* = 0.0368). SV^HEM^ had a linear correlation with SV^CM^ of 0.83. Bland Altman analysis for SV^CM^ had a bias of 2.28 with LOAs of − 24 and 29, a PE of 34%, and a RMSE of 13.88.

## Discussion/conclusions

This study describes a computer model estimation of the LVOTD which allows calculation of stroke volume and cardiac output with POC-TTE using only the LV VTI. Further we compare the accuracy of the computer model to two BSA-based methods of SV measurement. The data indicate that SV^cm^ shows the best correlation (0.87) with the lowest bias (5.2 ml) and lowest root mean square error (+/− 13 ml) relative to human expert measurement. However, the PE was 33%, which is greater than the 30% threshold commonly accepted for comparison with a pulmonary artery catheter (PAC).

There are several reasons automating LVOTD in the measurement of SV could improve POC-TTE. It would increase the yield of the metric, especially in difficult to image surgical patients [7]. It could also prove to make SV assessment more accurate because LVOTD measurement is difficult, prone to error, and the value is squared (SV = π (LVOT D/2)^2^ x LV VTI). This source of error is even more important if TTE is being performed by less expert providers.

Agreement between all 3 models of SV estimation show significant correlation using Bland-Altman analysis; the SV^CM^ model shows the best correlation, lowest bias, and the narrowest limits of agreement. When evaluated in a separate validation group, LVOTD^cm^ remains predictive. As shown in Fig. 3, the BSA-derived models have relatively good agreement at lower stroke volumes, but their variability increases dramatically as SV increases past normal values. SV^CM^ remains relatively constant throughout the values of SV.

While SV^cm^ is superior to BSA based methods it still has a PE of 33%, which is higher than the recommend threshold of 30%. Also, the root mean square error is +/− 13, which could be important in assessing metrics like stroke volume variation, but this is in line with the error reported in multiple studies comparing TTE to PAC in measurement of CO which range from 23 to 40% [7]. This study is describing one step in automating SV assessment with echo, further work is needed evaluate and improve the technique. At the very least it shows that it is possible to estimate the LVOT using readily available clinical data like gender, age, height and weight.

The inclusion of SV and CO in a POC-TTE makes echo an important tool in the management of shock in critically ill patients. Cardiac output combined with assessments of LV ejection fraction and right ventricular function help guide the use of inotropic support, while stroke volume is an important quantitative measure of volume status. Further if the mean arterial blood pressure is known the systemic vascular resistance can be estimated, allowing informed decision-making about systemic vasopressors [4, 5, 7, 8]. The study and other efforts to automate and make point of care TTE easier and more reproducible could have important effects on patient care and outcome.

With further testing and development, it is possible that SV^CM^ could become available at the bedside as a handheld app or embedded software on an ultrasound system. In the meantime, the SV cannot be assessed in about 30% of critically ill patients.

By obviating the need for LVOTD measurement, the derivation of a reliable estimator of LVOTD would increase the diagnostic yield of VR assessment by TTE, which is the most significant restraint on accurate estimation of cardiac function by echocardiography at the bedside. Also, once validated, it could reduce the error associated with human measurement. Where TTE is a useful adjunct in the assessment of the critically patient currently, this increased yield from roughly 70% to great than 90% would improve its usefulness to more patients and to the clinician. Currently, this derived model is the best estimator of LVOTD in the literature known to us.

Limitations of this research includes its retrospective design, use of a convenience sample, high rate of male patients expected in an urban trauma population, and skewed rates of minorities present in our patient population. The skewed sample of gender and race may limit its usefulness for some populations as there are differences in LV morphology between races, especially in response to hypertension [11], as well as difference normal cardiac morphology between genders [12]. Also, the intentional use of idealized images to optimize the model resulted in a lower yield of measurements then would be expected using clinical criteria thus creating selection bias.

This biased convenience sample of patients should be validated in future study utilizing an all-comer design. Further the model should be compared human expert measurement against a gold standard pulmonary artery catheter (PAC) in assessment of SV and CO.

Our computer derived model for LVOTD will allow for hemodynamic monitoring with comparable agreement to TTE performed by a human expert and superior estimation to two BSA methods. By requiring only measurement of LVOT VTI for accurate estimation of cardiac physiologic indices, this will increase the number of patients that can successfully be monitored by TTE by a bedside clinician, including those patients whose LVOTD images are inadequate or difficult to obtain. This increase in diagnostic yield could greatly increase the usefulness of TTE by improving accuracy, allowing serial measurement more readily, and allowing re-assessment of cardiac function following clinical interventions such as vasopressor or inotrope initiation, or volume resuscitation. Further validation of the model against the gold standard PAC and is needed.

## Data Availability

The datasets used and/or analyzed during the current study are available from the corresponding author on reasonable request.

## Abbreviations

BSA: Body surface area
CO: Cardiac output
EF: Ejection fraction
FREEs: Focused rapid echocardiographic evaluation
HEM: Human expert measurement
LOA: Limits of agreement
LVOTD: Left ventricular outflow tract diameter
LVOTD^BSA^: Left ventricular outflow tract diameter, body surface area
LVOTD^CM^: Left ventricular outflow tract diameter, computer model
LVOTD^HEM^: Left ventricular outflow tract diameter, human expert measurement
LVOTD^mBSA^: Left ventricular outflow tract, modified body surface area
mBSA: Modified body surface area
PE: Percent error
POC-TTE: Point-of-care transthoracic echocardiography
SV: Stroke volume
SV^BSA^: Stroke volume, body surface area
SV^CM^: Stroke volume, computer model
SV^HEM^: Stroke volume, human expert measurement
RMSE: Root mean square error
VTI: Velocity time integral
